# A Versatile and Reproducible Multi-Frequency Electrical Impedance Tomography System

**DOI:** 10.3390/s17020280

**Published:** 2017-01-31

**Authors:** James Avery, Thomas Dowrick, Mayo Faulkner, Nir Goren, David Holder

**Affiliations:** Department Medical Physics and Biomedical Engineering, University College London, London WC1E 6BT, UK; t.dowrick@ucl.ac.uk (T.D.); mayo.faulkner@ucl.ac.uk (M.F.); n.goren@ucl.ac.uk (N.G.); d.holder@ucl.ac.uk (D.H.)

**Keywords:** Electrical Impedance Tomography, brain imaging, long term monitoring, evoked activity, open source hardware, Arduino

## Abstract

A highly versatile Electrical Impedance Tomography (EIT) system, nicknamed the ScouseTom, has been developed. The system allows control over current amplitude, frequency, number of electrodes, injection protocol and data processing. Current is injected using a Keithley 6221 current source, and voltages are recorded with a 24-bit EEG system with minimum bandwidth of 3.2 kHz. Custom PCBs interface with a PC to control the measurement process, electrode addressing and triggering of external stimuli. The performance of the system was characterised using resistor phantoms to represent human scalp recordings, with an SNR of 77.5 dB, stable across a four hour recording and 20 Hz to 20 kHz. In studies of both haeomorrhage using scalp electrodes, and evoked activity using epicortical electrode mats in rats, it was possible to reconstruct images matching established literature at known areas of onset. Data collected using scalp electrode in humans matched known tissue impedance spectra and was stable over frequency. The experimental procedure is software controlled and is readily adaptable to new paradigms. Where possible, commercial or open-source components were used, to minimise the complexity in reproduction. The hardware designs and software for the system have been released under an open source licence, encouraging contributions and allowing for rapid replication.

## 1. Introduction

### 1.1. Electrical Impedance Tomography

Electrical Impedance Tomography (EIT) is a medical imaging technique which reconstructs the internal conductivity of an object from boundary voltage measurements [1]. Existing clinical uses of EIT include imaging the lung [2], liver [3] and breast [4]; commercial EIT systems are now available for clinical use. EIT of brain function and pathology is another active field of research, with development in applications including epilepsy [5,6], acute stroke [7], traumatic brain injury [8,9], evoked potentials (EPs) [10] and activity in peripheral nerves [11].

### 1.2. EIT Hardware

A typical EIT system comprises a current source, a voltage measurement unit, switching circuitry to address multiple electrodes, and a controller to automate the measurement process. A sinusoidal current of a defined amplitude and frequency is injected through a pair of electrodes, with voltages measured at all electrodes. Typically, a single “measurement” is defined as the demodulated amplitude of the voltage at a single electrode, averaged over a chosen number of sine wave periods. These voltage measurements can be obtained either serially, or in parallel on all electrodes. The process is repeated for a number of different pairs of injection electrodes, where the particular sequence is referred to as the “protocol”. The complete data set consists of *n* voltage measurements, equal to the number of injection pairs multiplied by the total number of electrodes. While it is possible for a single frame of data to be used to reconstruct a conductivity profile of the target object, it is more common to use the difference between two separate frames, producing an image of the change in conductivity.

Existing EIT systems include the KHU [12], fEITER [13], Dartmouth EIT System [14], Xian EIT system [15], Swisstom Pioneer Set (Swisstom AG, Switzerland) and systems previously developed in our laboratory at UCL [16,17]. Typically these systems have at least 32 electrodes with voltages recorded in parallel to increase frame rate, Table 1. Current is injected with a frequency range of 11 Hz to 1 MHz, with frequencies 10 kHz to 100 kHz most common. The SNR is usually characterised using the noise present in recordings in a resistor phantom or saline filled tank, with most systems capable of >80 dB in at least a subset of measurements. The maximum output impedance typically ranges from 100 kΩ to 1 MΩ.

With the exception of those developed at UCL, the systems described were built primarily for EIT imaging in the torso, with little emphasis placed on capturing the data required for EIT imaging of the brain. Typically, a greater number of electrodes 32–128 are employed in brain EIT applications, with current applied through irregularly spaced pairs of electrodes, Table 2. Particularly in epicortical experiments, the current injection protocol may need to be altered in situ, based on the positioning of the electrode mat on the cortex [18]. Therefore, the systems developed in the UCL laboratory have allowed for arbitrary pairs of electrodes to be addressed, controlled via software. Additionally, the neural signals of interest are several orders of magnitude smaller than those of typical thoracic EIT applications and require coherent averaging which is triggered either by an external stimuli, or additional physiological signal [16]. In general, the technical implementations of existing commercial EIT systems designed for thoracic imaging make translation to EIT of brain function difficult. EIT systems typically perform all demodulation in hardware, transmitting only the amplitude averaged over a chosen number of periods of the carrier frequency. Therefore, it is not possible to obtain a continuous impedance signal or record additional signals such as EEG, both of which are necessary for EIT of fast-neural activity [10]. Further, performing the signal processing in hardware severely limits the ability to adjust parameters such as carrier frequency, measurement speed, and filter bandwidth to meet the different application requirements.

### 1.3. EIT Applications

**Figure 1:**
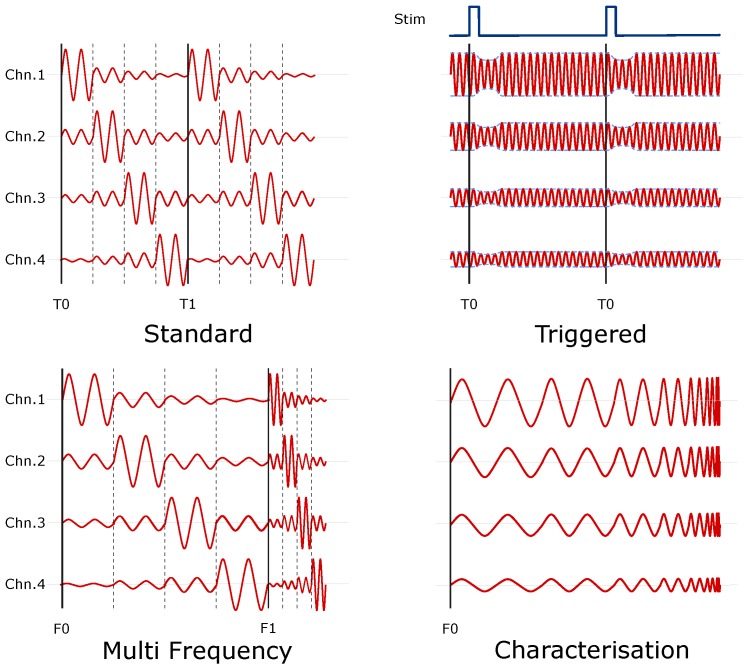
Modes of operation of the ScouseTom EIT system. Standard: Initial dataset collected at time T0, used as reference for subsequent datasets; Triggered: Current continuously injected simultaneous to multiple stimuli, with full dZ signal obtained through coherent averaging; MultiFrequency Data sets collected at multiple frequencies, with initial frequency F0 used as reference for subsequent measurements at higher frequencies; Characterisation Injection frequency is incremented across total range of interest.

Four main data collection strategies were identified for EIT experiments Figure 1. The most common method of data collection in EIT is time difference, where multiple data sets are obtained at different points in time, i.e., before and after the introduction of some perturbation, and the change in impedance over time is reconstructed. Such is the prevalence of this method, it can be considered the “standard” mode of operation, being used in the vast majority of successful experiments in the field [19].

The second method is time difference imaging, using an external triggered stimulus to allow for coherent averaging. Data collection is synchronised to an external stimulus (e.g., whisker stimulator in rats, or visual EPs in humans) which is also recorded. This method, first developed by Oh et al. [16] is used when impedance signals have low signal to noise ratio (SNR) and millisecond duration. Current is continuously injected, while the stimulus is repeated, generating multiple evoked potentials at different time points. Through coherent averaging, an impedance signal is obtained at each sample interval, yielding millisecond resolution.

In certain applications such as stroke [20] or breast imaging [4], where it is not possible to obtain a reference frame, the change in tissue impedance over frequency is instead reconstructed. To collect Multi frequency EIT data, a complete injection protocol is repeated for a number of frequencies, commonly 10 or 20, sufficient to capture the differences in tissue spectra [21]. The ordering of frequencies can be fixed, or randomised and the injection period of each particular frequency can be specified. Composite waveforms, consisting of multiple frequencies such as those implemented in the existing KHU [12] and UCL [17] systems, can also be generated. These methods are substantially less robust to modelling and instrumentation errors than standard EIT methods, and thus place more stringent requirements on the algorithms and hardware [22,23,24].

The final data collection method is a frequency sweep. While not directly used for EIT imaging, this method has previously been used to assist EIT experiments, allowing for impedance characterisation in-situ taking the place of a stand-alone impedance analyser [25] and selecting an optimal measurement frequency [26]. The injection frequency is incremented over a given range and step size with measurements taken at each point. Both increasing and decreasing sweeps are possible, as is randomised ordering.

### 1.4. Purpose

The motivation behind this work was to develop an EIT system that could be used primarily for imaging in the head and brain, while offering the maximum versatility, such that it could be easily reconfigured for different experimental methods. The following criteria was set:

- Arbitrary control over current amplitude, up to 10 mA and frequency in the kHz range. The current levels required for EIT can vary from tens of μA, for epicortical recordings in rats [16], to several mA when using human scalp electrodes [27].
- Parallel recording of all voltages for processing of data “offline”, allowing for additional signals, such as EEG/ECoG to be recorded alongside the EIT signal.
- Noise, frame rate and performance characteristics comparable to existing EIT systems. Target resistance changes of ≈0.1% have been previously identified for EIT recordings of fast neural activity related to neuronal depolarization and scalp recordings of epilepsy [6,16]. This requires stable current injection, <0.1% noise, and voltage recording with accuracy of ≈100 nV.
- Variable electrode count, up to a maximum of 256 electrodes.
- Ability to synchronise injection/recording with external triggers (whisker stimulation, visual, auditory). The phase of injected current should be randomised with respect to the stimulation, to minimise the phase related artefacts in EP recordings [28].
- Reconfigurable modes of operation, to allow for new functionality to be introduced as a later date. Ideally, this should be achievable through software or firmware changes only.
- Easily reproducible. Currently, construction of a non-commercial EIT system can take several months and existing publications on EIT systems typically lack enough detail to allow replication. A system which can be easily replicated using a mixture of off the shelf equipment, alongside open source software and hardware designs, will significantly reduce the workload and allow new systems to be assembled in a matter of weeks.

### 1.5. Experimental Design

Experiments were initially carried out on a resistor phantom to characterise the system across frequencies from 20 Hz to 20 kHz. Subsequently, the system was utilised in brain EIT applications, using all four data collection strategies outlined in Section 1.3 and the performance evaluated in each case. In some instances, the data were collected as part of other studies, where the main aim was successfully imaging the impedance change of interest [7,29]. In these cases additional analysis of the data was required to extract the relevant performance characteristics.

For the initial resistor phantom measurements, phantoms and measurement paradigms which represent realistic usage in brain EIT applications were chosen. The injected current complied with IEC 60601-1 [30] safety limits, the number of measurements and averaging times ≈100 ms were chosen to match those of real experiments. All recorded voltages were considered in subsequent analysis, with the exception of those with sufficiently low amplitude which are routinely neglected during reconstructions [31]. Thus the subsequent results are more representative of the performance of the system in an animal or human experiment, as opposed to the maximum achievable on a test bench. The characteristics of interest in these experiments were the noise and drift in measurements over time, and the reciprocity error (RE) the common metric for accuracy of EIT systems [12]. With a purely resistive test object, the amplitude of the measured voltages should be frequency independent. In reality, the components in any system will exhibit some variations with frequency which must be corrected for in post processing. Typically, measurements on a resistor phantom with known amplitude and phase are used to calculate the necessary calibration factors [12,17]. To study the frequency dependence of the ScouseTom, changes in amplitude across frequency were compared to the theoretical changes resulting from the anti-aliasing filters in two commercial EEG systems.

Data was collected in healthy human subjects using scalp EEG electrodes to represent the expected resting noise during clinical scalp EIT recordings [6,20,32]. Additionally, the suitability of the system for imaging impedance changes resulting from stroke during a feasibility study by Dowrick et al. [7] was assessed. The system was then utilised in measurements of evoked activity in the rat somatosensory cortex, using an established methodology [10,16]. Multi-frequency data were collected as part of a larger study in stroke patients in collaboration with University College London Hospital (UCLH). The noise performance of the system was evaluated across a frequency range previously used in a simulation study [33], with a subset of frequencies recorded for a longer time period to assess the degradation in data quality in real clinical measurements compared to those from a laboratory. Finally, measurements by Dowrick et al. [29] where the system was used in the impedance sweep mode of operation, where used to characterise the impedance of healthy and ischaemic rat brain in vivo.

## 2. Materials and Methods

### 2.1. System Design

**Figure 2:**
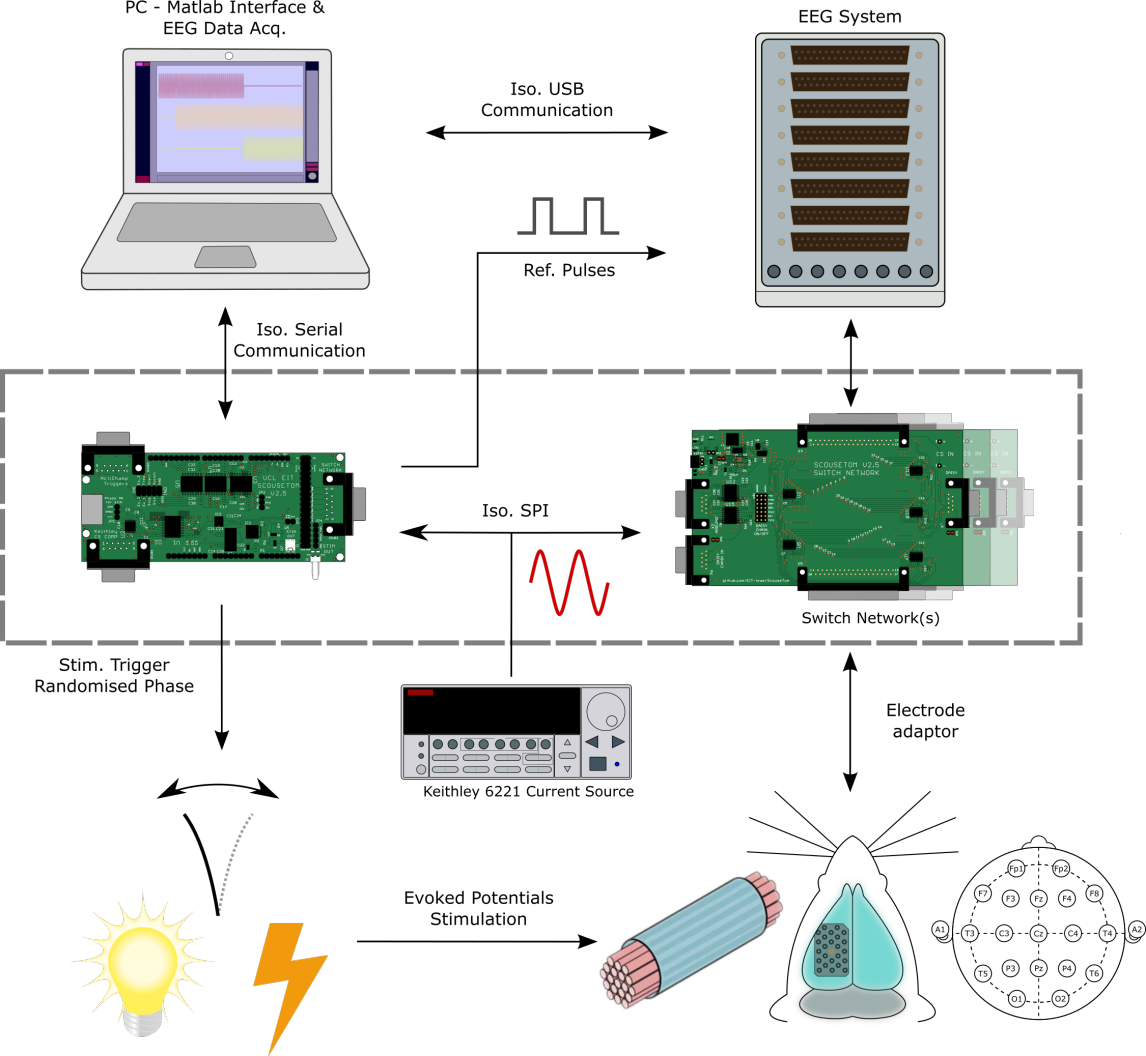
Overview of the ScouseTom system. The two bespoke PCBs (highlighted) control programming of the current source, multiplex the current injection channels and randomise the stimulation trigger with respect to the phase of the injected current. Voltages are recorded in parallel by the EEG system and stored on the PC for offline processing.

The ScouseTom EIT system, Figure 2, comprises a commercial current source and EEG amplifier, alongside custom switching/control circuitry and software. The system allows for software control over all aspects of the EIT measurement set-up: protocol, injection duration, frequency, amplitude. Time and frequency difference EIT data can be collected, at frequencies between 5 Hz and 20 kHz with a frame rate >100 per second achievable.

#### 2.1.1. Current Source

The Keithley 6221 current source (Keithley Instruments, Cleveland, OH, USA) is able to produce stable, low noise AC currents in the range 2 pA–100 mA, up to a frequency of 100 kHz; has a rated output impedance up to 1 GΩ, supports external triggering and phase marking, and is controllable via serial, USB and Ethernet connections. The versatility of the system as a whole is largely as a result of this wide operating range of the Keithley 6221. This range covers the majority of EIT applications, with the exception of those operating at high frequencies >100 kHz, such as breast imaging [14].

#### 2.1.2. Voltage Recording

The requirements for voltage recording are parallel data collection, low noise and the ability to save data for offline processing. EEG amplifiers offer an effective off the shelf solution with high performance systems such as the BioSemi ActiveTwo (Biosemi, Amsterdam, The Netherlands), actiCHamp (Brainproducts GmbH, Gilching, Germany) and g.tec HIamp (g.tec Medical Engineering GmbH, Graz, Austria) offering 24-bit resolution and a channel count up to 256. Each system offers a PC GUI for saving data to disk, data streaming over TCP/IP and the option to write custom software to interface with the device. These specifications come at the expense of maximum bandwidth, which in practice is limited by hardware anti-aliasing filters with typical cut off frequencies a tenth or a fifth of the sampling rate, Table 3. While this is sufficient for all brain EIT applications, Table 2, it may preclude use of the system in applications such as lung ventilation, which typically employ frequencies at 50 kHz or above [2]. For the experimental work presented here, either the BioSemi ActiveTwo (Biosemi, Amsterdam, the Netherlands) or actiCHamp (Brainproducts GmbH, Gilching, Germany) system was used for voltage recording.

#### 2.1.3. Controller and Switch Network

**Figure 3:**
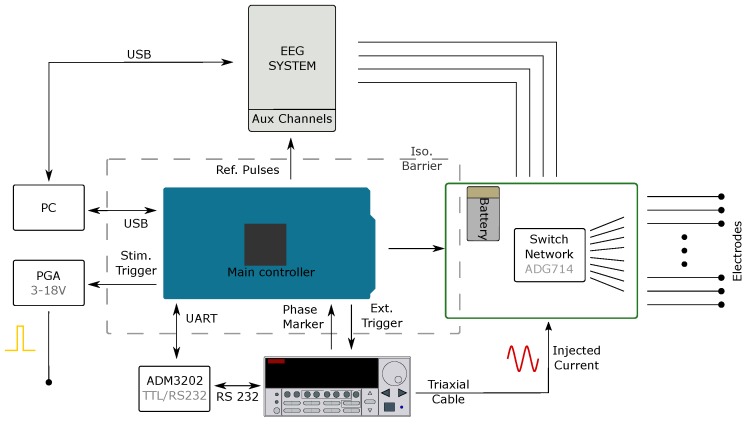
Overview of system architecture.

The system controller is based upon the Arduino development platform (Arduino Due-Arduino LLC) and two bespoke PCBs: a controller board or “shield” and a switch network board (highlighted in Figure 2). The controller shield contains the circuitry required for isolating the communication with other components of the system, namely RS232 and trigger-link connections with the current source, TTL with the EEG systems, and SPI connection to the switch networks. Due to the modular nature of the design and the variation of use cases, each connection was separately isolated from the mains supply, Figure 3, to ensure correct isolation regardless of experimental set-up.

The current source is programmed via RS232 by the controller, with external triggering and zero-phase marker signals transmitted via a trigger-link cable. The external triggering enables the phase of the injected current to be reset upon switching, or shorter injections to be retriggered by an external pulse. The pulses controlling the stimulation for use in Triggered-Averaging mode are sent by the Arduino controller, with user programmable width from 1.5 μs to >10 s. The time between these pulses is also set by the user, but the precise timing is modified by the controller following the suggestions in Aristovich et al. [28] to occur at a random time with respect to the phase of the injected current. This is achieved by offsetting the trigger pulse by a random phase with respect to the Zero-Phase Marker received from the Keithley 6221. Alongside this TTL trigger, a simultaneous stimulation pulse with a programmable range of 3 to 18 V can be sent for direct electrical stimulation.

Coded pulses sent by the controller are recorded by the EEG system and used as reference during data processing. Pulses are sent to indicate the start and stop of current injection, the switching of electrode pairs, changing of injected frequency, stimulation triggers and the out-of-compliance status of the current source.

The switch networks comprise two individual series of daisy chained ADG714 CMOS switches (Analog Devices, Norwood, MA, USA), one each for the source and sink connections from the current source. The switch networks themselves can be daisy chained together, enabling current injection between any two electrodes from a total of 128. The switchboard is battery powered, and communicates with the controller through a digitally isolated SPI bus, Figure 3. The possible time between switching electrode injection pairs ranges from 100 μs to approximately 70 min for 128 channels, which is more than sufficient to meet the frame rate requirements of all use cases.

#### 2.1.4. Controller Software

As the Arduino platform is not hardware specific, porting the software to another device with different architecture is comparatively simple, and does not constrain the system to a specific board in future iterations. Currently, the PC software for serial communication with the controller is written in MATLAB (The MathWorks Inc., Natick, MA, USA) but the commands can be easily replicated in another language.

#### 2.1.5. Data Processing Software

In conventional EIT systems, demodulation of the AC signal is performed in hardware, and only the averaged value for each short injection is transmitted and stored in the PC; the user has little control over the parameters used during processing. Whilst it is possible to alter the firmware for research devices such as the KHU [12] and UCLH [17], technical constraints such as on-board RAM and limited processing time impact the versatility of these systems.

As the ScouseTom system stores the continuous modulated voltages captured via an EEG system, the demodulation and data processing must be implemented in PC software, currently implemented in MATLAB. The software employs zero-phase IIR band pass filtering and the Hilbert transform to produce the envelope and phase of the amplitude modulated signal. The simultaneous EEG signal is obtained by low pass filtering the original signal. This also allows the user control over all the parameters during demodulation and averaging, demodulation method, centre frequency, bandwidth, filter type etc. This versatility is necessary to process data in EP studies, when the system is used in Triggered mode, as the measurement time is orders of magnitude longer than that of conventional EIT, and the synchronised EEG signal is essential.

#### 2.1.6. Electrode Connectors

Standard 9 and 37 D-Sub connectors are used for internal connections within the system. Custom connectors and PCBs were created to allow connection to a range of common electrode interfaces, including EEG electrodes, depth electrodes and Omnetics (Omnetics Connector Corp., Minneapolis, MN, USA) devices. Bespoke connectors and cabling can be added to the system for use with non-standard electrode arrays.

### 2.2. System Characterisation

To assess the noise and drift characteristics of the system, experiments were performed on the Cardiff phantom [34], configured as a purely resistive load.

#### 2.2.1. Resistor Phantom—Noise & Drift

A 100 μA injection current at 2 kHz was used, for 100 ms per measurement, over the course of four hours. Voltages were recorded with the BioSemi EEG system. The injection electrode protocol was one previously designed for recordings in the head, with 34 different injection pairs and 16 measurement electrodes, for a total of 544 voltage measurements [35]. This was reduced to 363 after rejection of measurements on injection electrodes and boundary voltages below 250 μV. The injection protocol was repeated every 3.4 s over the course of four hours, resulting in a total of 4235 frames.

As with other EIT systems the noise was considered across three time intervals: 100 frames (approximately five minutes) representing a typical short term measurement, and one and four hours [36]. These longer recordings replicate the use of the system in long term time difference recordings in monitoring applications [9,37] as well as in triggered mode during EP studies [10]. To evaluate the drift in performance over time, the change in voltages over the four hour recording was also calculated, as well as the SNR for each block of 100 frames. The reciprocity error (RE), where the ratio of the impedance measured with the voltage and current electrode pairs swapped, was calculated using the same 100 frames, and expressed as an absolute error in percentage.

#### 2.2.2. Resistor Phantom—Frequency Response

Data was collected at 15 frequencies between 20 Hz and 2 kHz using the BioSemi ActiveTwo and 33 frequencies between 20 Hz and 20 kHz for the actiCHamp system. To reduce the data collection time, six injection pairs from the protocol used previously were chosen, resulting in a total of 64 voltage measurements for analysis. The time per measurement was frequency dependent, equivalent to 32 sine-wave periods for frequencies below 200 Hz and 64 periods for those above. The whole protocol was repeated for 10 frames, with the frequency order randomised within each frame. The current amplitude was 100 μA for all frequencies. The voltages recorded were normalised with respect to the amplitude at 20 Hz, and expressed as a percentage. The corresponding amplitude decrease resulting from the anti-aliasing filters in both EEG systems was calculated for cut off frequencies quoted at 1.7 kHz and 7.5 kHz for the BioSemi and ActiCHamp respectively. To assess the noise frequency dependence of the noise performance, the SNR across all frequencies was also calculated.

### 2.3. Experimental Data

To evaluate the system experimentally and clinically, experiments were performed using each of the four modes of operation. All animal works undertaken in this or previous studies were approved by the UK Home Office and in accordance with its regulations (Project number: PPL 70/7450). Experiments on human subjects were approved either by the UCL REC, the local Research Ethics Committee and NHS/HSC R&D (IRAS ID: 168765). Certain methods were common across some experiments.

#### 2.3.1. Scalp Recordings—Preparation

All recordings on the human scalp were performed using 32 EEG electrodes (EasyCap, Herrsching, Germany), using the configuration described by Tidswell et al. [27], which includes 21 locations from the EEG 10–20 standard [38] and 11 additional electrodes. For these experiments, the locations were updated to match the nearest equivalents in either the 10–10 or 10–5 extensions [39]. Each electrode site was first cleaned with surgical spirit, then abraded using Nuprep gel (Weaver and Co., Aurora, CO, USA), with the electrode finally affixes using Elefix conductive paste (Nihon Kohden, Tokyo, Japan).

#### 2.3.2. EIT Reconstruction

EIT images were reconstructed using the same methodology used in other studies by the UCL group [7,10,18]. First the “forward problem” and sensitivity matrix was calculated using the Parallel EIT Solver [40], in a c. 4 million element tetrahedral mesh. The linear inverse solution was obtained using zeroth-order Tikhonov regularisation, with the hyperparameter *λ* chosen through cross-validation. The conductivity changes were reconstructed in a lower resolution hexahedral mesh of approximately 100,000 elements, and were subsequently post-processed using a noise based correction [10].

#### 2.3.3. Time Difference—Imaging Haemorrhage

“Standard” time difference EIT data were recorded in an anesthetised rat, from 40 spring-loaded gold plated electrodes placed on the skull in an even distribution. Intracerebral haemorrhage was produced by injection of 50 μL of autologous blood via a cannula into the brain at 5 μL per min [7]. Given the resistance of blood and grey matter at 1 kHz is approximately 1.4 $\Omega m^{-1}$ and 10 $\Omega m^{-1}$ respectively [25], this produced up to a seven-fold increase in conductivity localised around the injection site. A current of 100 μA at 2 kHz was injected, using a protocol of c. 60 injection pairs, with each injection lasting 1 s. The specific protocol was adapted for each individual experiment, dependent upon the quality of electrode contacts. A complete frame of EIT data was recorded every minute, over a total of 30 min, including a 10 min baseline prior to the injection of blood. Time difference images were reconstructed for every subsequent frame recorded after starting the injection of blood. This procedure was repeated in a total of 7 rats.

#### 2.3.4. Time Difference—Scalp Recordings

To assess the performance of the system in a clinically realistic scenario, EIT recordings were made in 10 healthy volunteers in a seated position. Current of 160 μA at 1.2 kHz was injected for 54 ms, equivalent to 64 sinewave periods, between 31 pairs of electrodes, resulting in a complete frame every 1.6 s. The injection pairs were chosen to maximise both the number of independent measurements and the overall magnitude of the voltages [33]. A complete frame consisted of a total of 930 measurements, 540 of which were considered in subsequent analysis, after rejecting measurements below 1 mV. A total of 60 frames were recorded over the course of 20 min for each subject.

#### 2.3.5. Triggered Averaging—Rat Somatosensory Cortex

The ScouseTom system was utilised in measurements of evoked activity using epicortical electrode arrays in anesthetised rats, repeating methodology from previous studies [5,10]. Arrays of platinised stainless-steel electrodes embedded in silicon, with 0.6 mm diameter contacts spaced 1.2 mm apart were placed directly onto the surface of the brain, targeting the somatosensory cortex. Whilst previous experiments had employed a single grid of 30 electrode contacts on a single side of the brain, in this study two grids of 57 electrodes were used, one on each hemisphere, for a total of 114. The activity was induced via electrical stimuli to the forepaw, delivered in 10 mA pulses of 1 ms duration at a frequency of 2 Hz, triggered by the ScouseTom controller. Current was injected at 1.7 kHz with 50 μA amplitude, for 30 s across a pair of electrodes for a total of 60 stimuli, with voltages on 114 electrodes recorded in parallel using the ActiChamp system with 25 kHz sampling rate. This process was repeated for c. 50 pairs of injection electrodes over the course of 25 min to produce a complete data set of c. 7000 voltages. The signals were demodulated with 1 kHz bandwidth, yielding 2 ms time resolution and coherent averaging was performed on 60 trials of 500 ms centered around the time of stimulation. EIT images were reconstructed at each 2 ms time point, using 10 ms prior to stimulation as the baseline. To allow for a comparison to previous studies with previous systems, the noise was also calculated after demodulation using a reduced bandwidth of 250 Hz [16].

#### 2.3.6. Multifrequency—Scalp Recordings

Multifrequency EIT data was collected as part of clinical trial in collaboration with the Hyper Acute Stroke unit (HASU) at University College London Hospital (UCLH). The injection protocol comprised the same 31 pairs used in previous scalp recordings, Section 2.3.4. Current was injected at 17 frequencies evenly spaced across the usable range of the BioSemi system, i.e., from 5 Hz to 2 kHz. As with previous multifrequency experiments, Section 2.2.2, the length of each injection was frequency dependent to reduce the total recording time. Current was injected for the equivalent of 32 periods at carrier frequencies below 200 Hz, 64 periods between 200 and 1 kHz, and 128 periods above 1 kHz. The amplitude of injected current varied with frequency as per the guidelines in IEC 60601 [30], except frequencies below 200 Hz which, as in previous studies [17], were reduced by half to ensure the current was not perceptible and to avoid saturation of the EEG amplifier due to the larger contact impedance at these frequencies. The protocol was repeated three times, taking a total of 20 min to complete. A further dataset using only three frequencies, 200 Hz, 1.2 kHz and 2 kHz, was also recorded on each patient, to better evaluate the frequency dependence of the noise and drift performance of the system. In this case, a total of 60 frames were collected over the course of 25 min. Data were collected in 23 patients, for a total of 31 datasets with 17 frequencies and 29 longer duration, 3 frequency recordings. After rejection of negligible channels, each dataset comprised 540 voltages per frequency.

#### 2.3.7. Impedance Spectrum Characterisation—Ischaemic Rat Brain

Impedance spectrum measurements were made with the ScouseTom system [29], in the kHz range on healthy (n = 112 voltage measurements in 4 rats) and ischaemic (n = 56 in 2 rats) rat brains. Using an 30 contact epicortical electrode array, Section 2.3.5. A current of 100 μA was injected through a single pair of electrodes, located on opposite corners of the array. The frequency of injected current was increased in 5 Hz intervals from 1 Hz to 100 Hz, 10 Hz intervals from 100 Hz to 1 kHz and 50 Hz intervals between 1 kHz and 3 kHz, for a total of 136, with a minimum of 50 periods of the waveform recorded at each frequency. This sweep was repeated in ascending and descending order and finally with random ordering. Voltages from each electrode were averaged together at each frequency. The relative change in impedance, rather than the absolute value, was calculated, by comparing the voltages at each frequency.

### 2.4. Data Presentation

For all experiments, a single “measurement” corresponds to the demodulated amplitude (modulus) at each carrier frequency. Noise and drift were calculated using all measurements except those with negligible boundary voltages (<250 μV), which were removed before processing. Unless stated otherwise, all data are presented as mean ± standard deviation across all voltage measurements and frames. Where applicable, the noise in repeated measurements is presented as three values, to aid comparisons to other systems in the literature: first as the standard deviation in absolute voltage, then as the ratio of the mean to standard deviation both as a percentage, and Signal-to-Noise ratio in dB. In accordance with previous studies, SNR was calculated differently for the triggered averaging experiment in the rat cortex. Instead the SNR was the ratio of the peak voltage change δz following stimulation to the standard deviation before stimulus [16]. The experimental set-up for each experiment is summarised in Table 4.

## 3. Results

### 3.1. Resistor Phantom—Noise & Drift

**Figure 4:**
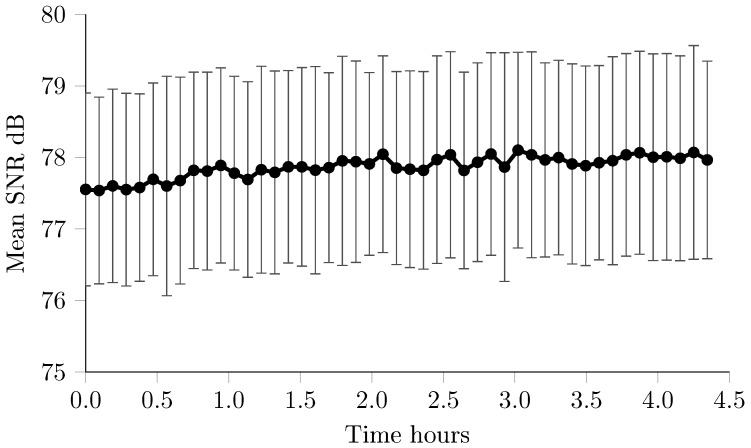
SNR per 100 frames (n = 363 measurements) over entire 4 h recording on Cardiff phantom.

The signal amplitude across all measurements and repeats was 2.66 mV ± 0.24 mV standard deviation. The noise in the first 100 frames was 0.356 μV ± 0.058 μV, equivalent to 0.013% ± 0.002% or 77.5 dB ± 1.3 dB SNR. The noise increased over the longer term, with 0.637 μV ± 0.064 μV or 0.024% ± 0.002% and 72.4 dB ± 0.66 dB for one hour, and 1.522 μV ± 0.205 μV or 0.17% ± 0.074% and 64.9 dB ± 1.24 dB and four hour recordings. As has been found with other EIT systems [36], this decrease in SNR was not observed when considering each consecutive block of 100 frames, Figure 4. Over the course of the four hour recording, the change in voltage was 5.61 μV ± 1.04 μV or 0.21% ± 0.04%. The reciprocity error (RE) was 0.42%.

### 3.2. Resistor Phantom—Frequency Response

**Figure 5:**
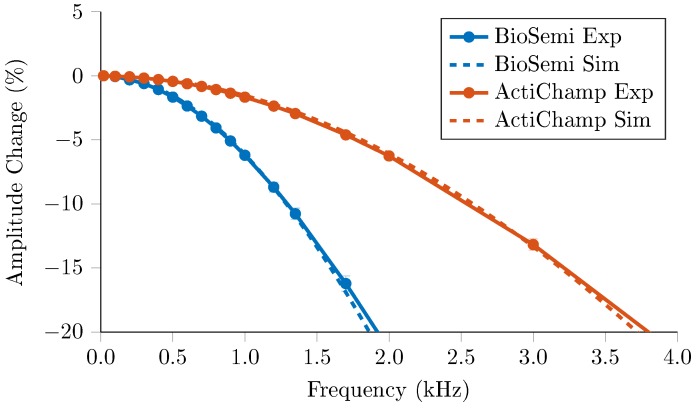
Decrease in amplitude across frequency as recorded with the BioSemi and ActiChamp systems (n = 64 measurements), compared to simulated changes from respective anti-aliasing filters.

In both systems, frequency response correlated with the gain of the respective filter, Figure 5, with a mean error of 0.07% and 0.09% for frequencies below the cut off for the BioSemi and actiCHamp respectively. The amplitude decrease was 1.86 mV or 21.0% from 20 Hz to 2 kHz for the BioSemi and 0.57 mV or 6.26% for the actiCHamp across the same range. Across the full 20 kHz range, the total decrease was 9.15 mV or 99.6%. Below these cut-off frequencies, there was no significant change in SNR across frequency, with 68.9 dB ± 9.42 dB for the BioSemi and 71.4 dB ± 12.6 dB for the actiCHamp.

### 3.3. Time Difference—Imaging Haemorrhage

**Figure 6:**
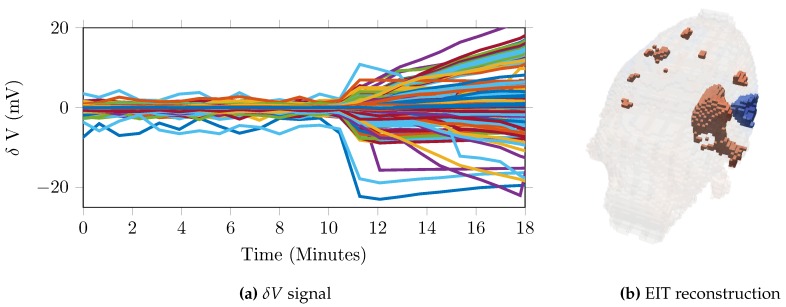
Example result from stroke experiment (**a**) recorded change in voltages (n = 1381) with haemorrhage onset at T = 10 min, (**b**) EIT reconstruction at T = 18 min.

The noise in the baseline recordings (n = 115 in N = 15) before intervention was 0.64 μV ± 2.12 μV equivalent to 1.90% ± 3.6% or 42.4 ± 11.9 dB. The SNR per experiment (N = 15) ranged from 28.4 to 62.1 dB. Over the 10 min injection period, changes of 20.6 mV ± 5.4 mV occurred (n = 7) Figure 6a, with a further increase of 2.2 mV ± 1.4 mV over the remaining measurement time. The time course of the impedance change was consistent with the injection period of the blood into the brain. Physiologically representative localised impedance changes could successfully be reconstructed in 5 out of the 7 experiments, Figure 6b.

### 3.4. Time Difference—Scalp Recordings

The signal amplitude across all subjects was 5.4 mV ± 1.97 mV and the drift during all recordings was 15.1 μV or 0.26%. The noise across all subjects was 37.2 μV ± 39.0 μV or 0.71% ± 0.65%. The noise in each subject ranged from 8.55 μV to 53.9 μV or 0.18% to 0.94%. The equivalent SNR overall was 46.5 dB ± 4.14 dB, ranging from 41.6 to 56.0 dB between subjects.

### 3.5. Triggered Averaging—Rat Somatosensory Cortex

**Figure 7:**
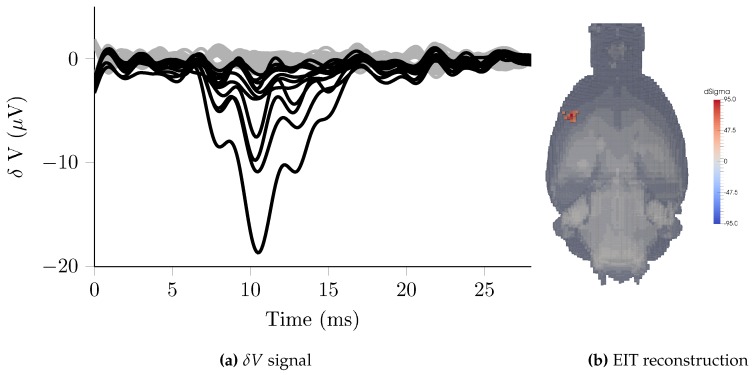
(**a**) Voltage changes recorded in rat somatosensory cortex during evoked potentials (n = 114) for a single pair of injecting electrodes. Channels with significant changes (*δV* > 3σ) are highlighted; (**b**) Example EIT image during electrical forepaw stimulation at T = 11 ms after stimulation.

The baseline noise was 0.37 μV ± 0.048 μV or 0.009% ± 0.005% of the baseline. Significant impedance changes (δV >3σ), a single injection pair example in Figure 7a, ranged from 1.20 μV to 18.7 μV (mean 3.97 μV), equivalent to 3.13 to 33.0 (mean 8.86) SNR. EIT reconstructions with this dataset show the onset at 7 ms approximately 1 mm below the surface within the primary somatosensory cortex (S1), before expanding to a larger volume reaching a maximum between 11–12 ms, Figure 7b, then spreading to adjacent areas and finally disappearing at approximately 18 ms. Using the reduced bandwidth of 250 Hz, the noise was 0.18 μV ± 0.06 μV or 0.004% ± 0.002% of the baseline.

### 3.6. Multifrequency—Scalp Recordings

**Figure 8:**
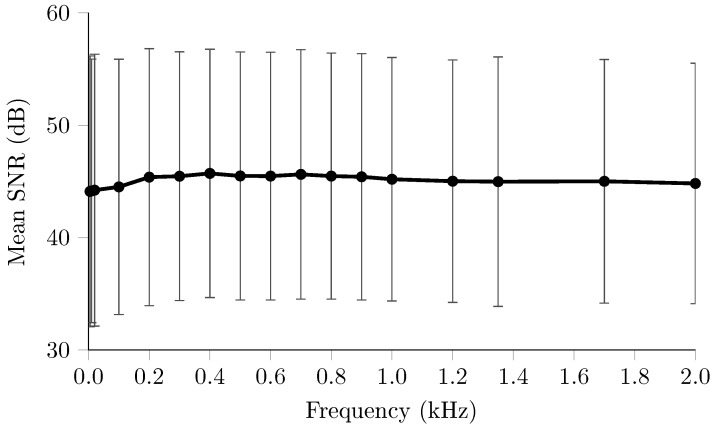
SNR of multi frequency scalp recordings on stroke patients (n = 540, N = 23).

The variation in SNR across frequency, Figure 8, was not significant (P<0.05), ranging from 44.1 to 45.5 dB. However, the reduction of current amplitude at frequencies below 200 Hz reduced the SNR by 1 dB compared to subsequent frequencies. The longer term recordings also did not demonstrate any significant frequency dependence (P<0.05) in SNR with 41.6 ± 7.9 dB, 41.4 ± 8.25 dB and 40.7 ± 8.2 dB for 0.2, 1.2 and 2 kHz respectively. Similarly the drifts of 0.74%, 0.61%, 0.68% were also not significantly variable across frequency.

### 3.7. Impedance Spectrum Measurement

**Figure 9:**
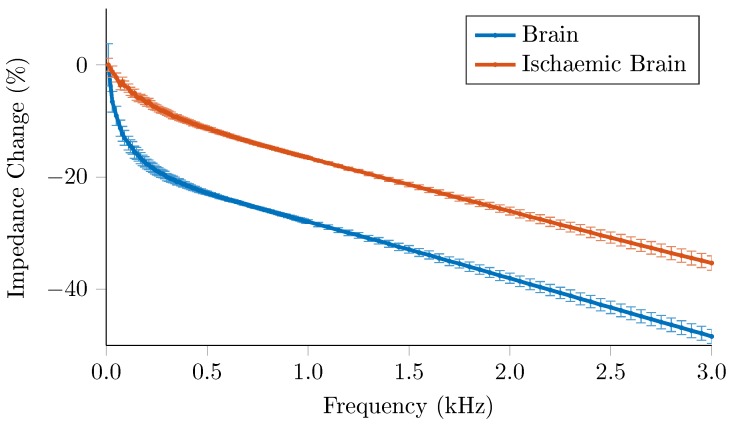
Impedance spectrum of healthy (n = 112, 4 rats) and ischaemic brain (n = 56, 2 rats) as measured with ScouseTom system. Error bars correspond to the standard error.

Healthy brain tissue showed a non linear decrease of 15% impedance over 0–250 Hz, with ischaemic brain showing a decrease of 7% over the same range, with a more linear slope Figure 9. Above 250 Hz, the impedance of both tissue types decreased at the same rate.

## 4. Discussion

### 4.1. System Characterisation

Results from the resistor phantom with realistic loads demonstrated a short term SNR of 77.5 dB ± 1.3 dB, with a reduction of 5 dB over an hour, and a total of 12.6 dB over the whole four hour period. As has been observed in existing EIT systems [36], this apparent decrease is as a result of low frequency drifts, likely from temperature changes in the current source, rather than degradation of the performance of the system, Figure 4. Overall, the SNR was lower than figures reported for other systems, which are capable of measuring with SNR above 90 dB [12,14]. The amplitude of the voltages recorded was 2.66 mV, orders of magnitude less than the input range of the EEG amplifier used (≈250 mV), and did not take full advantage of the high dynamic range offered by these amplifiers. The other EIT systems in the literature incorporate a programmable gain amplifier before digitisation to overcome this problem, often necessitating detailed calibration before data collection. It is therefore possible that certain measurements with larger injected currents and voltage amplitudes could far exceed the SNR reported in this study. One of the few systems in the literature which targeted the same frequency range and thus with the same constraints on current and voltage amplitude, demonstrated a noise of 0.1%, close to an order of magnitude greater than those reported here [17]. The reciprocity error (RE) of 0.42% is comparable to other EIT systems, despite the lack of calibration [12,14,36]. This is likely as a result of the comparatively low frequency used in this study, where the effect of stray capacitance in minimal.

The frequency response of the system as a whole matched that of the EEG systems used, and the SNR was consistent across frequency. This suggests that beyond correcting for the EEG amplifier gain, frequency specific calibration will not offer significant benefits to the accuracy of the system. If higher frequencies (>20 kHz) were recorded using a different EEG system, then stray capacitance could no longer be ignored, and calibration would likely be required.

### 4.2. Time Difference

The decrease in SNR in animal and human experiments is in agreement with previous studies [32] which demonstrated system noise accounts for approximately 5%, with physiological noise dominating. As with the long term resistor phantom recordings, the drifts across the recordings reduced the equivalent SNR. This physiological variability is evident in the range of SNR between subjects in both rat and human subjects. The system noise is thus masked by electrochemical or physiological changes over the course of the experiment. Despite this, conductivity changes resulting from the haemorrhage model could be imaged and correctly localised in the majority of cases. The SNR in the scalp recordings was decreased further in some recordings by artefacts resulting from subject or electrode movement. Between patients the SNR ranged from 41.6 to 56.0 dB, equivalent to 0.18% to 0.94%, which was less than the 2% observed by Romsauerova et al. [20] using a similar current amplitude and frequency, and within the 37.9 to 62.8 dB range observed by Xu et al. [41] using an order of magnitude greater amplitude at 50 kHz. Thus the ScouseTom system offers comparable or greater performance to existing systems, but with increased versatility in experimental settings.

### 4.3. Triggered Averaging

The baseline noise in these recordings agreed with that observed in previous fast-neural activity experiments which reported noise of 0.18 μV ± 0.04 μV, using a bandwidth of 250 Hz [16,42] and averaging over 120 repeated stimuli. However, the results presented in these studies were obtained after averaging of only 60 trials, suggesting a decrease in background noise when using the ScouseTom system. This not only offers benefits in terms of signal quality, but shorter data collection times reduce the effects of physiological drifts and allow for longer injection protocols, potentially improving EIT reconstructions.

Previous experiments with this methodology with mechanical stimulation of the rat whisker by Aristovich et al. [10] were found to be in good agreement with established literature and cross validated with optical imaging and depth probes both in initial onset [43], and spread of activity [44]. Whilst cross validation was not performed in this study, the literature is equally well established regarding forepaw stimulation and the location and depth of the area of onset in Figure 7b matches expectations [45,46,47]. Due to the high density of electrodes required, existing 32 channel systems limit measurements to a single cortical area on one side of the brain. The increased electrode count of the ScouseTom enables greater coverage of the cortex, as well as the possibility of simultaneous recording with additional depth electrodes.

### 4.4. Multifrequency

The stability of the system across frequency previously demonstrated in the resistor phantom experiments was also present in recordings in stroke patients, as SNR did not demonstrate a significant frequency dependence. However, overall the SNR did decrease compared to recordings in subjects at rest, Section 3.4, which were largely a result of a greater prevalence of motion artefacts from patient movement. The stability over frequency and increased electrode movement were further demonstrated in the drifts over the 25 min recordings, which were similar for all three frequencies. In simulation studies multifrequency methods have been shown to be robust to noise equivalent to 60–70 dB [23,24], 10–20 dB greater than those in both scalp experiments. However, the methods were shown to be highly sensitive to modelling errors such as electrode shape and position, and in particular systematic errors in impedance spectra of the tissues. These results suggest that SNR alone may not decide the success of imaging in these applications, and the highlight importance of the frequency invariant performance of ScouseTom system. Whilst the imaging in these applications is still the subject of research [24,48,49], the datasets collected with this system constitute the most comprehensive recorded and form a basis for future research.

### 4.5. Characterisation

The frequency sweep mode of operation is effectively that of a dedicated impedance analyser, with the flexibility of addressing any pair of electrodes for current injection and recording voltages on all electrodes in parallel. These results were consistent with previous studies in the area [50,51] and also allowed comparisons between two and four terminal measurements simultaneously.

### 4.6. Design Criteria and Technical Limitations

The design criteria set out initially were necessarily broad in order for the system to adapt to the four modes of operation successfully. The reconfigurability of the system meets the requirements for range of injected currents, electrode count, synchronisation and recording of simultaneous EEG signals. In doing so, the system has enabled experiments not previously possible, particularly when used in triggered averaging mode. The noise performance of the system was comparable to existing EIT systems, and was consistent across frequency. Whilst the <0.1% noise criteria was met in epicortical recordings, the noise in scalp recordings was closer to 0.65%, which may preclude imaging epileptic seizures using the system in its current form. However, use of higher frequencies would allow for higher currents to be used, as well as reducing in band physiological noise. Therefore, future experiments should be performed with higher bandwidth systems such as the actiCHamp or g.tec HIamp, with a higher carrier frequency.

The main limitation of the ScouseTom is the maximum usable frequency of 20 kHz imposed by the bandwidth of the EEG systems. This range is sufficient for EIT of fast-neural and stroke, where the impedance contrast is limited to frequencies <5 kHz [5,10,33]. This narrow range may not be suitable for other brain monitoring applications where carrier frequencies >50 kHz are used [6,8,9]. As this is largely to take advantage of the higher current amplitudes allowed by IEC 60601-1 [30], this does not preclude the use of the ScouseTom in these experiments but the necessary reduction in current amplitude would impact SNR. Currently, the majority of the data processing is not performed in “real time”, as with some commercially available systems. However, as most EEG recorders provide open source software, or allow streaming of data via TCP/IP, suitable software can be developed to allow for real time applications where needed.

### 4.7. Reproducibility and Recommendations for Use

All of the hardware designs, PCB layouts and associated software have been made available (see Appendix) to allow interested parties to replicate and modify the system. The ScouseTom system is quick to reproduce, as all but two components are commercially available, and the bespoke PCBs are simple compared to those used in other research EIT systems. Within the UCL group, assembly and testing of a new system once all the parts have been acquired can be performed with a week. The major expense is an appropriate EEG system ($21,000 for 32 channels), close to four times the cost of the current source and custom PCBs ($6000). However, the additional cost to researchers already performing electrophysiological experiments is minimal, as the system is compatible with most state-of-the-art EEG systems. Thus this system greatly reduces the cost and complexity for those interested in including impedance measurements into their experiments.

## 5. Conclusions

The ScouseTom, a new highly versatile EIT system has been utilised successfully in a range of experiments, particularly those involving EIT of the head or nerve. A key advantage of the system is the high level of control it offers over all aspects of the experimental process. This versatility is a result of the stable performance of the hardware across frequency and load, as well as adaptable modes of operation. The new system facilitated EIT experiments into time difference imaging of stroke, multi-frequency stroke type classification and stimulation-triggered evoked potentials. In all four modes of operation used, the performance of the system was comparable to or exceeded that of existing systems in the literature. By incorporating commercial and open-source hardware and software where possible, the complexity in reproducing the system is minimised. All relevant schematics and software are available on an open source license, with reproduction and contribution encouraged.

## Figures and Tables

**Table 1:** Existing EIT Systems.

System	Electrodes	Voltage Recording	Frequencies (Hz)	Frames per Second	Noise/SNR	Max Output Impedance (Ω)
KHU	16–64	Parallel	11–500 k	100	80 dB–120 dB	5.75 M
fEITER	32	Parallel	10 k	100	90 dB	-
Dartmouth	32	Parallel	1 k–1 M	100	80 dB–100 dB	100 k
Swisstom Pioneer Set	32	Parallel	50 k–250 k	50	-	-
Xian	>16	Sequential	1.6 k–380 k	-	>80 dB	2 M
UCL MK2.5	32	Sequential	20–256 k	26	80 dB	1 M

**Table 2:** Example of experimental requirements.

Mode	Frequency	Signal Bandwidth	Amplitude	Extra Data	Electrodes
Stroke & Head Injury	100 Hz–10 kHz	Frequency dependent	>1 mA	Voltage drift	32
Evoked Potentials	2 kHz	2 kHz	50 uA	CAP	128
Epilepsy	2 kHz	Slow—10 Hz; Fast—2 kHz	50 uA	EEG/ECoG	32–128
Nerve	5 kHz	3 kHz	50 uA	CAP	32

**Table 3:** Summary of EEG amplifier specifications.

	BioSemi	actiCHamp	g.tec HIamp
Max. Sampling Rate	16 kHz	100 kHz	38.4 kHz
Resolution	24-bit	24-bit	24-bit
Max. Channel Count	256	256	256
Input Range	±262 mV	±400 mV	±250 mV
Anti-aliasing	3.2 kHz	20 kHz/8 kHz *	19.2 kHz

* Anti-aliasing filter dependent on actiCHamp hardware version.

**Table 4:** Summary of experiments performed using the ScouseTom system.

	Experiment	Current μA	Frequencies (No.)	EEG System	Voltages	Frames
Resistor Phantom	System charac.	100	2 kHz	BioSemi	363	4235
	Frequency Response	100	20 Hz–2 kHz (15) & 20 Hz–20 kHz (15)	BioSemi & actiCHamp	64	10
Time Difference	Stroke	100	2 kHz	BioSemi	418–1381	10 (ref) 6–50 (stroke)
	Scalp	160	1.2 kHz	BioSemi	540	60
Triggered Averaging	Evoked Potentials	50	1.7 kHz	actiCHamp	5088	N/A 500 ms trial
Multi Frequency	Scalp	45–280	5 Hz–2 kHz (17)	BioSemi	540	3
	Scalp Long term	90–280	200 Hz–2 kHz (3)	BioSemi	540	60
Charac.	Ischaemic Brain	100	1 Hz–3 kHz (136)	BioSemi	112	3

## Appendix A. Hardware & Software Resources

All of the hardware and software is released under a GNU General Public License v3.0, with contribution and distribution welcomed. Controller hardware schematics, PCBs and firmware, along with 3D printable case models and documentation available at https://github.com/EIT-team/ScouseTom. Software for processing data available at https://github.com/EIT-team/Load_data.

## References

[1] Metherall P., Barber D., Smallwood R., Brown B. (1996). Three dimensional electrical impedance tomography. Nature.

[2] Frerichs I. (2000). Electrical impedance tomography (EIT) in applications related to lung and ventilation: A review of experimental and clinical activities. Physiol. Meas..

[3] You F., Shuai W., Shi X., Fu F., Liu R., Dong X., Dössel O., Schlegel W.C. (2009). In vivo Monitoring by EIT for the Pig’s Bleeding after Liver Injury. World Congress on Medical Physics and Biomedical Engineering, September 7–12, 2009, Munich, Germany: Vol. 25/2 Diagnostic Imaging.

[4] Halter R.J., Hartov A., Paulsen K.D. (2008). A broadband high-frequency electrical impedance tomography system for breast imaging. IEEE Trans. Biomed. Eng..

[5] Vongerichten A.N., dos Santos G.S., Aristovich K., Avery J., McEvoy A., Walker M., Holder D.S. (2016). Characterisation and imaging of cortical impedance changes during interictal and ictal activity in the anaesthetised rat. NeuroImage.

[6] Fabrizi L., Sparkes M., Horesh L., Abascal J.F.P.-J., McEwan A., Bayford R.H., Elwes R., Binnie C.D., Holder D.S. (2006). Factors limiting the application of electrical impedance tomography for identification of regional conductivity changes using scalp electrodes during epileptic seizures in humans. Physiol. Meas..

[7] Dowrick T., Blochet C., Holder D. (2016). In vivo bioimpedance changes during haemorrhagic and ischaemic stroke in rats: Towards 3D stroke imaging using electrical impedance tomography. Physiol. Meas..

[8] Manwaring P.K., Moodie K.L., Hartov A., Manwaring K.H., Halter R.J. (2013). Intracranial electrical impedance tomography: A method of continuous monitoring in an animal model of head trauma. Anesth. Analg..

[9] Fu F., Li B., Dai M., Hu S.J., Li X., Xu C.H., Wang B., Yang B., Tang M.X., Dong X.Z. (2014). Use of electrical impedance tomography to monitor regional cerebral edema during clinical dehydration treatment. PLoS ONE.

[10] Aristovich K.Y., Packham B.C., Koo H., dos Santos G.S., McEvoy A., Holder D.S. (2016). Imaging fast electrical activity in the brain with electrical impedance tomography. NeuroImage.

[11] Aristovich K., Blochet C., Avery J., Donega M., Holder D. EIT of evoked and spontaneous activity in peripheral nerve. Proceedings of the 17th International Conference on Biomedical Applications of Electrical Impedance Tomography.

[12] Wi H., Sohal H., McEwan A.L., Woo E.J., Oh T.I. (2014). Multi-Frequency Electrical Impedance Tomography System With Automatic Self-Calibration for Long-Term Monitoring. IEEE Trans. Biomed. Circuits Syst..

[13] McCann H., Ahsan S.T., Davidson J.L., Robinson R.L., Wright P., Pomfrett C.J.D. A portable instrument for high-speed brain function imaging: FEITER. Proceedings of the 2011 IEEE Annual International Conference on Engineering in Medicine and Biology Society (EMBC).

[14] Khan S., Manwaring P., Borsic A., Halter R. (2015). FPGA-based voltage and current dual drive system for high frame rate electrical impedance tomography. IEEE Trans. Med. Imaging.

[15] Shi X., Xiuzhen D., You F., Fu F., Liu R. High precision Multifrequency Electrical Impedance Tomography System and Preliminary imaging results on saline tank. Proceedings of the 27th Annual International Conference of the IEEE Engineering in Medicine and Biology Society.

[16] Oh T., Gilad O., Ghosh A., Schuettler M., Holder D.S. (2011). A novel method for recording neuronal depolarization with recording at 125–825 Hz: Implications for imaging fast neural activity in the brain with electrical impedance tomography. Med. Biol. Eng. Comput..

[17] McEwan A., Romsauerova A., Yerworth R., Horesh L., Bayford R., Holder D. (2006). Design and calibration of a compact multi-frequency EIT system for acute stroke imaging. Physiol. Meas..

[18] Aristovich K.Y., dos Santos G.S., Packham B.C., Holder D.S. (2014). A method for reconstructing tomographic images of evoked neural activity with electrical impedance tomography using intracranial planar arrays. Physiol. Meas..

[19] Bayford R., Tizzard A. (2012). Bioimpedance imaging: An overview of potential clinical applications. Analyst.

[20] Romsauerova A., McEwan A., Horesh L., Yerworth R., Bayford R.H., Holder D.S. (2006). Multi-frequency electrical impedance tomography (EIT) of the adult human head: Initial findings in brain tumours, arteriovenous malformations and chronic stroke, development of an analysis method and calibration. Physiol. Meas..

[21] Ammari H., Garnier J., Giovangigli L., Jing W., Seo J.K. (2016). Spectroscopic imaging of a dilute cell suspension. J. Math. Pures Appl..

[22] Ahn S., Oh T.I., Jun S.C., Seo J.K., Woo E.J. (2011). Validation of weighted frequency-difference EIT using a three-dimensional hemisphere model and phantom. Physiol. Meas..

[23] Malone E., dos Santos G.S., Holder D., Arridge S. (2014). Multifrequency electrical impedance tomography using spectral constraints. IEEE Trans. Med. Imaging.

[24] Alberti G.S., Ammari H., Jin B., Seo J.K., Zhang W. (2016). The Linearized Inverse Problem in Multifrequency Electrical Impedance Tomography. SIAM J. Imaging Sci..

[25] Gabriel C., Peyman A., Grant E.H. (2009). Electrical conductivity of tissue at frequencies below 1 MHz. Phys. Med. Biol..

[26] Vongerichten A., Santos G., Aristovich K., Holder D. Impedance changes during evoked responses in the rat cortex in the 225–1575 Hz frequency range. Proceedings of the XVth International Conference of Electrical Bioimpedance, XIV Conference on Electrical Impedance Tomography.

[27] Tidswell T., Gibson A., Bayford R.H., Holder D.S. (2001). Three-dimensional electrical impedance tomography of human brain activity. NeuroImage.

[28] Aristovich K.Y., Dos Santos G.S., Holder D.S. (2015). Investigation of potential artefactual changes in measurements of impedance changes during evoked activity: Implications to electrical impedance tomography of brain function. Physiol. Meas..

[29] Dowrick T., Blochet C., Holder D. (2015). In vivo bioimpedance measurement of healthy and ischaemic rat brain: Implications for stroke imaging using electrical impedance tomography. Physiol. Meas..

[30] International Electrotechnical Commission (2002). IEC 60601-1 Medical Electrical Equipment: Part 1: General Requirements for Basic Safety and Essential Performance.

[31] Packham B., Koo H., Romsauerova A., Ahn S., McEwan A., Jun S., Holder D. (2012). Comparison of frequency difference reconstruction algorithms for the detection of acute stroke using EIT in a realistic head-shaped tank. Physiol. Meas..

[32] Fabrizi L., McEwan A., Woo E., Holder D.S. (2007). Analysis of resting noise characteristics of three EIT systems in order to compare suitability for time difference imaging with scalp electrodes during epileptic seizures. Physiol. Meas..

[33] Malone E., Jehl M., Arridge S., Betcke T., Holder D. (2014). Stroke type differentiation using spectrally constrained multifrequency EIT: Evaluation of feasibility in a realistic head model. Physiol. Meas..

[34] Griffiths H. (1995). A Cole phantom for EIT. Physiol. Meas..

[35] Fabrizi L., McEwan A., Oh T., Woo E.J., Holder D.S. (2009). An electrode addressing protocol for imaging brain function with electrical impedance tomography using a 16-channel semi-parallel system. Physiol. Meas..

[36] Oh T.I., Woo E.J., Holder D. (2007). Multi-frequency EIT system with radially symmetric architecture: KHU Mark1. Physiol. Meas..

[37] Adler A., Amato M.B., Arnold J.H., Bayford R., Bodenstein M., Böhm S.H., Brown B.H., Frerichs I., Stenqvist O., Weiler N. (2012). Whither lung EIT: Where are we, where do we want to go and what do we need to get there?. Physiol. Meas..

[38] Jasper H. (1958). Report of the committee on methods of clinical examination in electroencephalography. Electroencephalogr. Clin. Neurophysiol..

[39] Oostenveld R., Praamstra P. (2001). The five percent electrode system for high-resolution EEG and ERP measurements. Clin. Neurophysiol..

[40] Jehl M., Dedner A., Betcke T., Aristovich K., Klofkorn R., Holder D. (2014). A Fast Parallel Solver for the Forward Problem in Electrical Impedance Tomography. IEEE Trans. Bio-Med. Eng..

[41] Xu S., Dai M., Xu C., Chen C., Tang M., Shi X., Dong X. (2011). Performance evaluation of five types of Ag/AgCl bio-electrodes for cerebral electrical impedance tomography. Ann. Biomed. Eng..

[42] Packham B., Barnes G., Sato G., Santos D., Aristovich K., Gilad O., Ghosh A., Oh T., Holder D. (2016). Empirical validation of statistical parametric mapping for group imaging of fast neural activity using electrical impedance tomography. Physiol. Meas..

[43] Armstrong-James M., Callahan C.A., Friedman M.A. (1991). Thalamo-cortical processing of vibrissal information in the rat. I. Intracortical origins of surround but not centre-receptive fields of layer IV neurones in the rat S1 barrel field cortex. J. Comp. Neurol..

[44] Petersen C.C. (2007). The functional organization of the barrel cortex. Neuron.

[45] Peeters R., Tindemans I., De Schutter E., Van der Linden A. (2001). Comparing BOLD fMRI signal changes in the awake and anesthetized rat during electrical forepaw stimulation. Magn. Reson. Imaging.

[46] Masamoto K., Kim T., Fukuda M., Wang P., Kim S.G. (2007). Relationship between neural, vascular, and BOLD signals in isoflurane-anesthetized rat somatosensory cortex. Cereb. Cortex.

[47] Lowe A.S., Beech J.S., Williams S.C. (2007). Small animal, whole brain fMRI: Innocuous and nociceptive forepaw stimulation. Neuroimage.

[48] Malone E., dos Santos G.S., Holder D., Arridge S. (2015). A reconstruction-classification method for multifrequency electrical impedance tomography. IEEE Trans. Med. Imaging.

[49] Jang J., Seo J. (2015). Detection of admittivity anomaly on high-contrast heterogeneous backgrounds using frequency difference EIT. Physiol. Meas..

[50] Ranck J.B. (1963). Analysis of specific impedance of rabbit cerebral cortex. Exp. Neurol..

[51] Logothetis N.K., Kayser C., Oeltermann A. (2007). In Vivo Measurement of Cortical Impedance Spectrum in Monkeys: Implications for Signal Propagation. Neuron.

